# (2*Z*)-1-(5-Hy­droxy-3-methyl-1-phenyl-1*H*-pyrazol-4-yl)-3-(4-methyl­anilino)­but-2-en-1-one

**DOI:** 10.1107/S1600536812006526

**Published:** 2012-02-24

**Authors:** Abdullah M. Asiri, Abdulrahman O. Al-Youbi, Seik Weng Ng, Edward R. T. Tiekink

**Affiliations:** a Chemistry Department, Faculty of Science, King Abdulaziz University, PO Box 80203, Jeddah, Saudi Arabia; b The Center of Excellence for Advanced Materials Research, King Abdulaziz University, Jeddah, PO Box 80203, Saudi Arabia; c Department of Chemistry, University of Malaya, 50603 Kuala Lumpur, Malaysia

## Abstract

A twist is evident in the title compound, C_21_H_21_N_3_O_2_, the dihedral angle between the terminal six-membered rings being 29.46 (10)°; the linked five- and six-membered rings are coplanar [1.30 (11)°]. The carbonyl O atom accepts intra­molecular hydrogen bonds from the adjacent hy­droxy and amine groups. The three-dimensional crystal packing is achieved through C—H⋯π inter­actions.

## Related literature

For background to the synthesis, see: Gelin *et al.* (1983 ▶); Bendaas *et al.* (1999 ▶). For the structures of the 4-chloro and 4-meth­oxy analogues, see: Asiri, Al-Youbi, Alamry *et al.* (2011 ▶); Asiri, Al-Youbi, Faidallah *et al.* (2011 ▶).
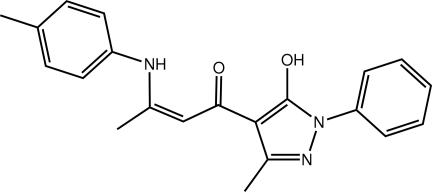


## Experimental

### 

#### Crystal data

C_21_H_21_N_3_O_2_
*M* *_r_* = 347.41
Monoclinic, 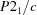

*a* = 14.9041 (8) Å
*b* = 6.9222 (4) Å
*c* = 17.1921 (8) Å
β = 96.190 (5)°
*V* = 1763.35 (16) Å^3^
*Z* = 4
Mo *K*α radiation
μ = 0.09 mm^−1^
*T* = 100 K
0.40 × 0.02 × 0.02 mm

#### Data collection

Agilent SuperNova Dual diffractometer with an Atlas detector
Absorption correction: multi-scan (*CrysAlis PRO*; Agilent, 2011 ▶) *T*_min_ = 0.967, *T*_max_ = 0.998
12780 measured reflections
4055 independent reflections
2455 reflections with *I* > 2σ(*I*)
*R*_int_ = 0.076

#### Refinement

*R*[*F* ^2^ > 2σ(*F* ^2^)] = 0.060
*wR*(*F* ^2^) = 0.164
*S* = 1.02
4055 reflections
246 parameters
2 restraints
H atoms treated by a mixture of independent and constrained refinement
Δρ_max_ = 0.24 e Å^−3^
Δρ_min_ = −0.29 e Å^−3^

### 

Data collection: *CrysAlis PRO* (Agilent, 2011 ▶); cell refinement: *CrysAlis PRO*; data reduction: *CrysAlis PRO*; program(s) used to solve structure: *SHELXS97* (Sheldrick, 2008 ▶); program(s) used to refine structure: *SHELXL97* (Sheldrick, 2008 ▶); molecular graphics: *ORTEP-3* (Farrugia, 1997 ▶) and *DIAMOND* (Brandenburg, 2006 ▶); software used to prepare material for publication: *publCIF* (Westrip, 2010 ▶).

## Supplementary Material

Crystal structure: contains datablock(s) global, I. DOI: 10.1107/S1600536812006526/hg5179sup1.cif

Structure factors: contains datablock(s) I. DOI: 10.1107/S1600536812006526/hg5179Isup2.hkl

Supplementary material file. DOI: 10.1107/S1600536812006526/hg5179Isup3.cml

Additional supplementary materials:  crystallographic information; 3D view; checkCIF report

## Figures and Tables

**Table 1:** Hydrogen-bond geometry (Å, °) *Cg*1 and *Cg*2 are the centroids of the C1–C6 and C15–C20 rings, respectively.

*D*—H⋯*A*	*D*—H	H⋯*A*	*D*⋯*A*	*D*—H⋯*A*
O1—H1⋯O2	0.86 (1)	1.71 (2)	2.509 (2)	155 (4)
N3—H2⋯O2	0.89 (1)	1.91 (2)	2.671 (3)	143 (2)
C14—H14*A*⋯*Cg*1^i^	0.98	2.69	3.475 (2)	138
C14—H14*C*⋯*Cg*1^ii^	0.98	2.66	3.563 (2)	153
C17—H17⋯*Cg*2^iii^	0.95	2.58	3.424 (2)	148

Symmetry codes: (i) 
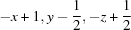
; (ii) 
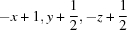
; (iii) 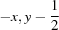, 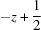.

**Fig. 1.**
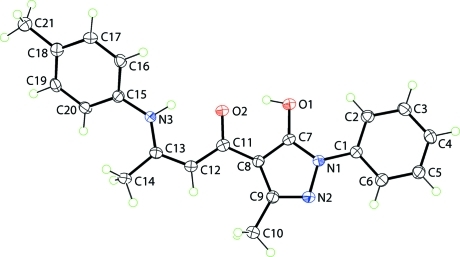
The molecular structure of (I) showing the atom-labelling scheme and displacement ellipsoids at the 50% probability level.

**Fig. 2.**
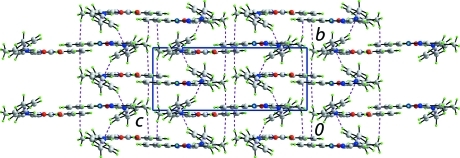
A view in projection down the a axis of the unit-cell contents of (I). The C—H···π interactions are shown as purple dashed lines.

## supplementary crystallographic information

### Comment

In connection with recent structural studies (Asiri, Al-Youbi, Alamry *et al.*, 2011; (Asiri, Al-Youbi, Faidallah *et al.*, 2011) of compounds prepared by reactions between pyrazoles and aniline derivatives following literature procedures (Gelin *et al.*, 1983; Bendaas *et al.*, 1999), the title compound, 3-(4-toluidino)-1-(5-hydroxy-3-methyl-1-phenyl-1*H*-pyrazol-4-yl)but-2-en-1-one (I) was investigated.

While in (I), Fig. 1, the linked five- and six-membered rings are co-planar, forming a dihedral angle of 1.30 (11)°, there is a twist about the N3—C15 bond as seen in the value of the C13—N3—C15—C16 torsion angle of 146.9 (2)°; the dihedral angle between the terminal six-membered rings is 29.46 (10)°. The carbonyl-O2 atom accepts hydrogen bonds from the adjacent hydroxyl- and amine-groups, Table 1. These groups do not participate in intermolecular interactions. Rather, molecules are consolidated in the three-dimensional crystal packing by C—H···π interactions, Fig. 2 and Table 1.

### Experimental

A solution of 4-acetoacetyl-5-hydroxy-3-methyl-1-phenylpyrazole (0.005 mol) and *p*-toluidine (0.005 mol) in ethanol (25 ml) was refluxed for 2 h. The precipitate, obtained from the hot solution, was collected, washed with methanol and recrystallized from its ethanol-benzene solution as yellow needles; *M*.pt: 419–421 K.

### Refinement

Carbon-bound H-atoms were placed in calculated positions [C—H = 0.95 to 0.98 Å, *U*_iso_(H) = 1.2 to 1.5*U*_eq_(C)] and were included in the refinement in the riding model approximation. The N—H and O—H-atoms were located in a difference Fourier map, and were refined with distance restraints of N—H = 0.88±0.01 and O—H = 0.84±0.01 Å, respectively; their *U*_iso_ values were refined. Owing to poor agreement, the (3 6 3) reflection was omitted from the final cycles of refinement.

### Crystal data

**Table:**

C_21_H_21_N_3_O_2_	*F*(000) = 736
*M**_r_* = 347.41	*D*_x_ = 1.309 Mg m^−^^3^
Monoclinic, *P*2_1_/*c*	Mo *K*α radiation, λ = 0.71073 Å
Hall symbol: -P 2ybc	Cell parameters from 2466 reflections
*a* = 14.9041 (8) Å	θ = 2.4–27.5°
*b* = 6.9222 (4) Å	µ = 0.09 mm^−^^1^
*c* = 17.1921 (8) Å	*T* = 100 K
β = 96.190 (5)°	Needle, yellow
*V* = 1763.35 (16) Å^3^	0.40 × 0.02 × 0.02 mm
*Z* = 4	

### Data collection

**Table:**

Agilent SuperNova Dual diffractometer with an Atlas detector	4055 independent reflections
Radiation source: SuperNova (Mo) X-ray Source	2455 reflections with *I* > 2σ(*I*)
Mirror monochromator	*R*_int_ = 0.076
Detector resolution: 10.4041 pixels mm^-1^	θ_max_ = 27.6°, θ_min_ = 2.4°
ω scan	*h* = −18→19
Absorption correction: multi-scan (*CrysAlis PRO*; Agilent, 2011)	*k* = −8→8
*T*_min_ = 0.967, *T*_max_ = 0.998	*l* = −22→18
12780 measured reflections	

### Refinement

**Table:**

Refinement on *F*^2^	Primary atom site location: structure-invariant direct methods
Least-squares matrix: full	Secondary atom site location: difference Fourier map
*R*[*F*^2^ > 2σ(*F*^2^)] = 0.060	Hydrogen site location: inferred from neighbouring sites
*wR*(*F*^2^) = 0.164	H atoms treated by a mixture of independent and constrained refinement
*S* = 1.02	*w* = 1/[σ^2^(*F*_o_^2^) + (0.065*P*)^2^ + 0.099*P*] where *P* = (*F*_o_^2^ + 2*F*_c_^2^)/3
4055 reflections	(Δ/σ)_max_ = 0.001
246 parameters	Δρ_max_ = 0.24 e Å^−^^3^
2 restraints	Δρ_min_ = −0.29 e Å^−^^3^

### Fractional atomic coordinates and isotropic or equivalent isotropic displacement parameters (Å^2^)

**Table:**

	*x*	*y*	*z*	*U*_iso_*/*U*_eq_	
O1	0.50304 (12)	0.0677 (2)	0.39888 (10)	0.0275 (4)	
O2	0.37132 (11)	0.0655 (2)	0.29485 (9)	0.0244 (4)	
N1	0.64285 (13)	0.0686 (2)	0.34357 (10)	0.0192 (4)	
N2	0.67114 (13)	0.0711 (2)	0.26853 (10)	0.0229 (4)	
N3	0.23145 (13)	0.0409 (3)	0.18426 (11)	0.0215 (4)	
C1	0.70881 (15)	0.0615 (3)	0.40934 (12)	0.0186 (5)	
C2	0.68417 (16)	0.0546 (3)	0.48530 (13)	0.0231 (5)	
H2A	0.6223	0.0541	0.4941	0.028*	
C3	0.75125 (17)	0.0486 (3)	0.54797 (13)	0.0256 (5)	
H3	0.7347	0.0431	0.5998	0.031*	
C4	0.84175 (17)	0.0503 (3)	0.53633 (13)	0.0257 (5)	
H4	0.8870	0.0465	0.5797	0.031*	
C5	0.86567 (17)	0.0577 (3)	0.46044 (13)	0.0245 (5)	
H5	0.9276	0.0600	0.4519	0.029*	
C6	0.79969 (15)	0.0618 (3)	0.39707 (13)	0.0227 (5)	
H6	0.8165	0.0649	0.3453	0.027*	
C7	0.55156 (15)	0.0701 (3)	0.33854 (13)	0.0193 (5)	
C8	0.51783 (15)	0.0733 (3)	0.25960 (12)	0.0187 (5)	
C9	0.59647 (16)	0.0733 (3)	0.21952 (13)	0.0215 (5)	
C10	0.60368 (17)	0.0740 (3)	0.13342 (13)	0.0302 (6)	
H10A	0.6674	0.0793	0.1243	0.045*	
H10B	0.5721	0.1871	0.1096	0.045*	
H10C	0.5763	−0.0439	0.1100	0.045*	
C11	0.42170 (16)	0.0712 (3)	0.23786 (13)	0.0207 (5)	
C12	0.38378 (16)	0.0712 (3)	0.15824 (12)	0.0205 (5)	
H12	0.4245	0.0831	0.1196	0.025*	
C13	0.29317 (16)	0.0556 (3)	0.13212 (12)	0.0200 (5)	
C14	0.26394 (16)	0.0493 (3)	0.04612 (12)	0.0229 (5)	
H14A	0.2209	−0.0566	0.0348	0.034*	
H14B	0.3167	0.0283	0.0177	0.034*	
H14C	0.2351	0.1720	0.0296	0.034*	
C15	0.13787 (16)	0.0037 (3)	0.17314 (13)	0.0209 (5)	
C16	0.10125 (15)	−0.1006 (3)	0.23183 (12)	0.0225 (5)	
H16	0.1400	−0.1484	0.2751	0.027*	
C17	0.00953 (16)	−0.1349 (3)	0.22785 (13)	0.0231 (5)	
H17	−0.0138	−0.2033	0.2692	0.028*	
C18	−0.04959 (16)	−0.0719 (3)	0.16470 (13)	0.0227 (5)	
C19	−0.01234 (16)	0.0307 (3)	0.10594 (13)	0.0229 (5)	
H19	−0.0509	0.0746	0.0618	0.028*	
C20	0.07922 (16)	0.0702 (3)	0.11024 (12)	0.0227 (5)	
H20	0.1022	0.1432	0.0700	0.027*	
C21	−0.14922 (16)	−0.1052 (3)	0.16189 (14)	0.0295 (6)	
H21	−0.1795	−0.0515	0.1132	0.044*	
H21B	−0.1721	−0.0416	0.2067	0.044*	
H21C	−0.1612	−0.2442	0.1639	0.044*	
H1	0.4494 (12)	0.069 (5)	0.375 (2)	0.102 (15)*	
H2	0.2582 (15)	0.034 (3)	0.2329 (7)	0.031 (7)*	

### Atomic displacement parameters (Å^2^)

**Table:**

	*U*^11^	*U*^22^	*U*^33^	*U*^12^	*U*^13^	*U*^23^
O1	0.0212 (10)	0.0405 (10)	0.0217 (9)	−0.0003 (8)	0.0060 (8)	−0.0002 (7)
O2	0.0207 (9)	0.0336 (9)	0.0199 (8)	−0.0008 (7)	0.0060 (7)	0.0005 (6)
N1	0.0186 (10)	0.0209 (9)	0.0184 (9)	0.0007 (7)	0.0039 (8)	−0.0005 (7)
N2	0.0219 (11)	0.0283 (10)	0.0190 (10)	−0.0012 (8)	0.0048 (8)	−0.0007 (7)
N3	0.0180 (11)	0.0307 (11)	0.0159 (10)	−0.0003 (8)	0.0016 (8)	0.0020 (8)
C1	0.0204 (12)	0.0138 (10)	0.0216 (11)	0.0004 (9)	0.0024 (9)	0.0017 (8)
C2	0.0235 (13)	0.0237 (11)	0.0224 (12)	0.0006 (10)	0.0039 (10)	−0.0017 (9)
C3	0.0288 (14)	0.0278 (12)	0.0203 (12)	0.0009 (10)	0.0028 (10)	−0.0003 (9)
C4	0.0277 (14)	0.0269 (12)	0.0213 (12)	−0.0019 (10)	−0.0024 (10)	−0.0005 (9)
C5	0.0224 (13)	0.0253 (12)	0.0256 (12)	−0.0007 (10)	0.0022 (10)	0.0008 (9)
C6	0.0218 (13)	0.0208 (11)	0.0254 (12)	−0.0010 (9)	0.0029 (10)	−0.0005 (9)
C7	0.0182 (12)	0.0175 (10)	0.0230 (12)	−0.0001 (9)	0.0058 (9)	−0.0005 (8)
C8	0.0184 (12)	0.0172 (10)	0.0203 (11)	−0.0008 (9)	0.0018 (9)	0.0009 (8)
C9	0.0218 (13)	0.0209 (11)	0.0218 (12)	−0.0001 (9)	0.0022 (10)	0.0009 (8)
C10	0.0248 (14)	0.0447 (15)	0.0214 (12)	0.0012 (11)	0.0038 (11)	0.0016 (10)
C11	0.0219 (13)	0.0164 (10)	0.0244 (12)	0.0002 (9)	0.0046 (10)	0.0007 (8)
C12	0.0216 (12)	0.0208 (11)	0.0196 (11)	−0.0004 (9)	0.0047 (9)	0.0009 (8)
C13	0.0230 (13)	0.0171 (10)	0.0202 (11)	0.0002 (9)	0.0043 (10)	0.0013 (8)
C14	0.0249 (13)	0.0261 (12)	0.0187 (11)	0.0002 (10)	0.0067 (10)	0.0002 (9)
C15	0.0195 (13)	0.0227 (11)	0.0205 (11)	−0.0008 (9)	0.0026 (9)	−0.0036 (8)
C16	0.0218 (13)	0.0237 (12)	0.0214 (12)	0.0037 (9)	0.0000 (10)	0.0018 (8)
C17	0.0250 (13)	0.0234 (11)	0.0219 (12)	−0.0010 (10)	0.0070 (10)	0.0026 (9)
C18	0.0226 (13)	0.0217 (11)	0.0239 (12)	0.0032 (9)	0.0026 (10)	−0.0041 (9)
C19	0.0232 (13)	0.0270 (12)	0.0176 (11)	0.0038 (9)	−0.0022 (10)	−0.0030 (8)
C20	0.0247 (13)	0.0249 (11)	0.0185 (11)	0.0027 (10)	0.0029 (10)	0.0019 (9)
C21	0.0260 (14)	0.0347 (13)	0.0279 (13)	−0.0015 (11)	0.0038 (11)	−0.0017 (10)

### Geometric parameters (Å, º)

**Table:**

O1—C7	1.327 (3)	C10—H10A	0.9800
O1—H1	0.858 (10)	C10—H10B	0.9800
O2—C11	1.298 (3)	C10—H10C	0.9800
N1—C7	1.354 (3)	C11—C12	1.423 (3)
N1—N2	1.400 (2)	C12—C13	1.381 (3)
N1—C1	1.417 (3)	C12—H12	0.9500
N2—C9	1.322 (3)	C13—C14	1.496 (3)
N3—C13	1.356 (3)	C14—H14A	0.9800
N3—C15	1.411 (3)	C14—H14B	0.9800
N3—H2	0.888 (10)	C14—H14C	0.9800
C1—C6	1.393 (3)	C15—C20	1.393 (3)
C1—C2	1.395 (3)	C15—C16	1.398 (3)
C2—C3	1.389 (3)	C16—C17	1.382 (3)
C2—H2A	0.9500	C16—H16	0.9500
C3—C4	1.385 (3)	C17—C18	1.393 (3)
C3—H3	0.9500	C17—H17	0.9500
C4—C5	1.390 (3)	C18—C19	1.398 (3)
C4—H4	0.9500	C18—C21	1.498 (3)
C5—C6	1.387 (3)	C19—C20	1.386 (3)
C5—H5	0.9500	C19—H19	0.9500
C6—H6	0.9500	C20—H20	0.9500
C7—C8	1.396 (3)	C21—H21	0.9800
C8—C9	1.422 (3)	C21—H21B	0.9800
C8—C11	1.441 (3)	C21—H21C	0.9800
C9—C10	1.496 (3)		
			
C7—O1—H1	101 (3)	H10B—C10—H10C	109.5
C7—N1—N2	109.97 (18)	O2—C11—C12	121.6 (2)
C7—N1—C1	131.08 (18)	O2—C11—C8	116.39 (19)
N2—N1—C1	118.94 (18)	C12—C11—C8	122.0 (2)
C9—N2—N1	105.72 (18)	C13—C12—C11	125.9 (2)
C13—N3—C15	130.94 (19)	C13—C12—H12	117.1
C13—N3—H2	111.0 (16)	C11—C12—H12	117.1
C15—N3—H2	117.1 (16)	N3—C13—C12	120.0 (2)
C6—C1—C2	120.0 (2)	N3—C13—C14	120.3 (2)
C6—C1—N1	118.77 (19)	C12—C13—C14	119.61 (19)
C2—C1—N1	121.2 (2)	C13—C14—H14A	109.5
C3—C2—C1	119.1 (2)	C13—C14—H14B	109.5
C3—C2—H2A	120.4	H14A—C14—H14B	109.5
C1—C2—H2A	120.4	C13—C14—H14C	109.5
C4—C3—C2	121.2 (2)	H14A—C14—H14C	109.5
C4—C3—H3	119.4	H14B—C14—H14C	109.5
C2—C3—H3	119.4	C20—C15—C16	118.0 (2)
C3—C4—C5	119.2 (2)	C20—C15—N3	125.0 (2)
C3—C4—H4	120.4	C16—C15—N3	117.0 (2)
C5—C4—H4	120.4	C17—C16—C15	120.9 (2)
C6—C5—C4	120.4 (2)	C17—C16—H16	119.5
C6—C5—H5	119.8	C15—C16—H16	119.5
C4—C5—H5	119.8	C16—C17—C18	121.6 (2)
C5—C6—C1	120.0 (2)	C16—C17—H17	119.2
C5—C6—H6	120.0	C18—C17—H17	119.2
C1—C6—H6	120.0	C17—C18—C19	117.1 (2)
O1—C7—N1	125.3 (2)	C17—C18—C21	121.2 (2)
O1—C7—C8	126.2 (2)	C19—C18—C21	121.6 (2)
N1—C7—C8	108.45 (19)	C20—C19—C18	121.8 (2)
C7—C8—C9	104.0 (2)	C20—C19—H19	119.1
C7—C8—C11	119.7 (2)	C18—C19—H19	119.1
C9—C8—C11	136.3 (2)	C19—C20—C15	120.5 (2)
N2—C9—C8	111.87 (19)	C19—C20—H20	119.7
N2—C9—C10	119.0 (2)	C15—C20—H20	119.7
C8—C9—C10	129.1 (2)	C18—C21—H21	109.5
C9—C10—H10A	109.5	C18—C21—H21B	109.5
C9—C10—H10B	109.5	H21—C21—H21B	109.5
H10A—C10—H10B	109.5	C18—C21—H21C	109.5
C9—C10—H10C	109.5	H21—C21—H21C	109.5
H10A—C10—H10C	109.5	H21B—C21—H21C	109.5
			
C7—N1—N2—C9	−0.2 (2)	C11—C8—C9—N2	−178.4 (2)
C1—N1—N2—C9	178.60 (16)	C7—C8—C9—C10	179.3 (2)
C7—N1—C1—C6	−180.0 (2)	C11—C8—C9—C10	1.1 (4)
N2—N1—C1—C6	1.5 (3)	C7—C8—C11—O2	−0.3 (3)
C7—N1—C1—C2	−0.1 (3)	C9—C8—C11—O2	177.8 (2)
N2—N1—C1—C2	−178.62 (17)	C7—C8—C11—C12	−179.00 (18)
C6—C1—C2—C3	0.1 (3)	C9—C8—C11—C12	−1.0 (4)
N1—C1—C2—C3	−179.74 (17)	O2—C11—C12—C13	−3.5 (3)
C1—C2—C3—C4	0.4 (3)	C8—C11—C12—C13	175.14 (19)
C2—C3—C4—C5	−0.2 (3)	C15—N3—C13—C12	−172.5 (2)
C3—C4—C5—C6	−0.5 (3)	C15—N3—C13—C14	5.7 (3)
C4—C5—C6—C1	1.0 (3)	C11—C12—C13—N3	0.9 (3)
C2—C1—C6—C5	−0.8 (3)	C11—C12—C13—C14	−177.35 (18)
N1—C1—C6—C5	179.08 (17)	C13—N3—C15—C20	−36.1 (3)
N2—N1—C7—O1	179.73 (18)	C13—N3—C15—C16	146.9 (2)
C1—N1—C7—O1	1.1 (3)	C20—C15—C16—C17	−0.6 (3)
N2—N1—C7—C8	0.1 (2)	N3—C15—C16—C17	176.58 (19)
C1—N1—C7—C8	−178.55 (18)	C15—C16—C17—C18	1.6 (3)
O1—C7—C8—C9	−179.57 (19)	C16—C17—C18—C19	−1.0 (3)
N1—C7—C8—C9	0.1 (2)	C16—C17—C18—C21	−178.35 (19)
O1—C7—C8—C11	−1.0 (3)	C17—C18—C19—C20	−0.6 (3)
N1—C7—C8—C11	178.65 (17)	C21—C18—C19—C20	176.7 (2)
N1—N2—C9—C8	0.3 (2)	C18—C19—C20—C15	1.7 (3)
N1—N2—C9—C10	−179.30 (17)	C16—C15—C20—C19	−1.0 (3)
C7—C8—C9—N2	−0.2 (2)	N3—C15—C20—C19	−177.94 (19)

### Hydrogen-bond geometry (Å, º)

Cg1 and Cg2 are the centroids of the C1–C6 and C15–C20 rings, respectively.

**Table:**

*D*—H···*A*	*D*—H	H···*A*	*D*···*A*	*D*—H···*A*
O1—H1···O2	0.86 (1)	1.71 (2)	2.509 (2)	155 (4)
N3—H2···O2	0.89 (1)	1.91 (2)	2.671 (3)	143 (2)
C14—H14*A*···*Cg*2^i^	0.98	2.69	3.475 (2)	138
C14—H14*C*···*Cg*2^ii^	0.98	2.66	3.563 (2)	153
C17—H17···*Cg*3^iii^	0.95	2.58	3.424 (2)	148

Symmetry codes: (i) −*x*+1, *y*−1/2, −*z*+1/2; (ii) −*x*+1, *y*+1/2, −*z*+1/2; (iii) −*x*, *y*−1/2, −*z*+1/2.
